# Pregnancy-Associated Plasma Protein (PAPP)-A2 in Physiology and Disease

**DOI:** 10.3390/cells10123576

**Published:** 2021-12-18

**Authors:** Vicente Barrios, Julie A. Chowen, Álvaro Martín-Rivada, Santiago Guerra-Cantera, Jesús Pozo, Shoshana Yakar, Ron G. Rosenfeld, Luis A. Pérez-Jurado, Juan Suárez, Jesús Argente

**Affiliations:** 1 Departments of Pediatrics & Pediatric Endocrinology, Hospital Infantil Universitario Niño Jesús, 28009 Madrid, Spain; vicente.barriossa@salud.madrid.org (V.B.); jachowen@gmail.com (J.A.C.); amartinrivada@gmail.com (Á.M.-R.); santiguerra8@gmail.com (S.G.-C.); jesuspozoroman@gmail.com (J.P.); 2 La Princesa Research Institute, 28009 Madrid, Spain; 3 Centro de Investigación Biomédica en Red de Fisiopatología de la Obesidad y Nutriciόn (CIBEROBN), Instituto de Salud Carlos III, 28029 Madrid, Spain; 4 IMDEA Food Institute, CEI UAM & CSIC, 28049 Madrid, Spain; 5 Department of Pediatrics, Universidad Autónoma de Madrid, 28049 Madrid, Spain; 6 Department of Molecular Pathobiology, David B. Kriser Dental Center, New York University College of Dentistry, New York, NY 10010, USA; sy1007@nyu.edu; 7 Department of Pediatrics, Oregon Health & Science University, Portland, OR 97239, USA; stat5consulting@yahoo.com; 8 Genetics Unit, Department of Experimental and Health Sciences, Universitat Pompeu Fabra, 08003 Barcelona, Spain; luis.perez@upf.edu; 9 Service of Genetics, Hospital del Mar and Hospital del Mar Research Institute (IMIM), 08003 Barcelona, Spain; 10 Centro de Investigación Biomédica en Red de Enfermedades Raras (CIBERER), Instituto de Salud Carlos III, 08036 Barcelona, Spain; 11 Departamento de Anatomía Humana, Medicina Legal e Historia de la Ciencia, Universidad de Málaga, 29071 Málaga, Spain; 12 Instituto de Investigación Biomédica de Málaga (IBIMA), 29010 Málaga, Spain

**Keywords:** growth hormone axis, IGF1, IGF2, IGFBPs, PAPP-A, PAPP-A2, STC1, STC2

## Abstract

The growth hormone (GH)/insulin-like growth factor (IGF) axis plays fundamental roles during development, maturation, and aging. Members of this axis, composed of various ligands, receptors, and binding proteins, are regulated in a tissue- and time-specific manner that requires precise control that is not completely understood. Some of the most recent advances in understanding the implications of this axis in human growth are derived from the identifications of new mutations in the gene encoding the pregnancy-associated plasma protein PAPP-A2 protease that liberates IGFs from their carrier proteins in a selective manner to allow binding to the IGF receptor 1. The identification of three nonrelated families with mutations in the *PAPP-A2* gene has shed light on how this protease affects human physiology. This review summarizes our understanding of the implications of PAPP-A2 in growth physiology, obtained from studies in genetically modified animal models and the PAPP-A2 deficient patients known to date.

## 1. Introduction

Longitudinal bone growth is governed by chondrocyte multiplication, hypertrophy of chondrocytes and ossification, with cartilage being replaced by mineralized bone. These processes are modulated by various factors, including growth hormone (GH) and insulin-like growth factor (IGF)1 [1], which act in both an endocrine and autocrine paracrine manner during prenatal, but especially postnatal growth. Indeed, the GH/IGF axis is responsible for prenatal and especially postnatal growth, with the expression levels, interactions and effects of the different members of this system changing to promote efficient growth, development and maintenance at all physiological stages.

**Figure 1:**
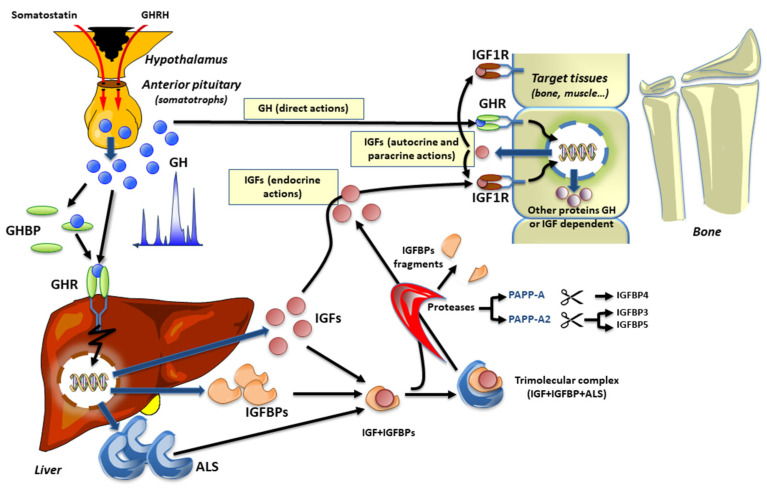
The GH-IGF axis and its endocrine, autocrine, and paracrine actions.

The hypothalamic-pituitary GH/IGF axis is regulated by the hypothalamic GH releasing hormone (GHRH) that binds its cognate receptor (GHRHR) in the anterior pituitary to stimulate GH secretion, which ultimately reaches the circulation and the target tissues. Ghrelin also plays an important role in the regulation of GH secretion, with its receptor being expressed in the pituitary gland, as well as in the hypothalamus, but at lower levels [2,3], and the lack of this receptor affects postnatal linear growth [4]. Through binding to the GH receptor (GHR) in the liver and other tissues, GH regulates liver production and secretion of IGF1. It should also be kept in mind that the production of IGF1 is also dependent on an adequate supply of glucose and amino acids, as well as lipids [5], thus representing a link between nutrition and growth. Glucose could control hepatic GH receptor levels, whereas certain amino acids appear to influence GH-stimulated IGF1 expression [6]. Once in circulation, IGF1 and IGF2, are bound to IGF binding proteins (IGFBPs) 1–6 in binary complexes. The high affinity IGFBP3 and IGFBP5 can also bind to a non-IGFBP component, the acid-labile subunit (ALS), to form a ternary complex that increases the half-life of IGF1 in the circulation. Indeed, the major role of the high-affinity IGFBPs is to modulate the bioavailability of the IGFs. The binding of GH to its receptor, GHR, activates Janus kinase (JAK) 2 and signal transducer and activator of transcription (STAT) 5 that mediate IGF1 synthesis in the liver. Importantly, GH regulates not only liver production of IGF1 but also the expression of the IGFBPs and the ALS, as well as inducing specific effects in other GH target tissues. Liberating the IGFs from their binding proteins in close proximity to the IGF1 receptor (IGF1R), brings to the GH/IGF axis another level of complexity, which includes specific proteases (Figure 1) that we review in detail below.

Other possible participants in this system have been proposed and include the insulin receptor (IR), the hybrid IR-IGF1 receptor (IGF1R) and various low-affinity IGF binding proteins. However, whether these latter proteins modulate the actions of the IGFs has not been reliably demonstrated and they may have different physiological actions from those attributed to IGFBP1–6; thus, they are now called IGFBP-related proteins [7].

The discovery, characterization, and advances in our understanding of the physiological mechanisms involved in growth have transpired over the past 60 years. The somatomedin hypothesis [8], which postulated that the actions of GH were mediated by a serum factor capable of incorporating sulfate into cartilage, was published in 1957. A few years later, the presence of serum peptides with growth-promoting actions and potent insulin-like metabolic actions was detected. These factors, with a molecular weight between 5–8 kDa, did not cause hypoglycemia despite their high concentrations in serum. This apparent paradox was resolved when this somatomedin activity was isolated by chromatography at a molecular weight ranging between 30–40 kDa, which was higher than previously described. Subsequently, the presence of binding proteins of these growth promoting factors was demonstrated [9]. This discovery helped to explain the normoglycemia that was observed, even with high circulating levels of these somatomedin factors, as these small peptides, later named IGF1 and IGF2, were shown to be bound to serum transport proteins resulting in the inhibition of their actions. It is also of note that insulin was shown not to bind to these proteins.

GH is secreted in a pulsatile manner from the somatotrophs of the anterior pituitary. In the circulation, GH can be found free or bound to GH binding proteins (GHBP). GH can activate the GH receptor (GHR) directly on target issues (“direct actions of GH”) or stimulate the production of ALS, IGFBPs, and IGFs, which also participate in the promotion of growth. To avoid the occurrence of hypoglycemia, IGFs circulate primarily bound to ALS and either IGFBP3 or IGFBP5 (ternary complex of 150 kDa). Specific proteases, such as PAPP-A and PAPP-A2, selectively exert proteolytic activity on IGFBP4 or IGFBP3 and IGFBP5, respectively. Subsequently, free IGF1 can activate its receptor on target tissues (“endocrine, autocrine, or paracrine actions” of IGF1). The red arrows for both somatostatin and GHRH indicate the release of these peptides in the hypothalamic-pituitary portal vasculature.

Most IGF1 actions on longitudinal growth are mediated through IGF1R and subsequent activation of its downstream mediators, mainly extracellular signal-regulated kinase (ERK) and phosphatidylinositol 3 kinase/Akt [10]. The IGF2R, in addition to binding mannose-6-phosphate (M6P) containing proteins, is responsible for an IGF2 degradation pathway and is also suggested to be involved in intracellular signal transduction, with IGF2R activation being linked with G-protein dependent effectors, such as ERK and protein kinase C [11]. Although the signaling capacity of IGF2R has been questioned, soluble M6P/IGF2R is important in growth and development via both IGF2 dependent and independent mechanisms. It is suggested that the precise function of the M6P/IGF2 receptor may vary depending on the ligands and/or cell type under consideration, but although this remains controversial, the M6P/IGF2R clearly plays a regulatory role in normal mammalian embryonic development.

The actions of IGFs are controlled by changes in their concentrations and that of their receptors, as well as modifications in their bioavailability. This bioavailability, or their capacity to bind and activate their receptors, is controlled by IGFBPs, regulate their distribution to the target tissues, enhance their half-life by protecting them from degradation, and can also potentiate their actions [12]. Approximately 75–80% of circulating IGFs is found in ternary complexes with IGFBP3 or IGFBP5 and the ALS, which augments the half-life of these growth factors to almost 16 h. Twenty percent of the IGFs are bound to binary complexes with IGFBPs, while less than 1% are present as free/unbound IGFs that have a half-life of around 10 min [13]. Numerous studies have shown that besides protecting IGFs, the IGFBPs have IGF independent effects on cell homeostasis, including growth, autophagy and angiogenesis, among others through binding to cell surface receptors and activating different signaling pathways, with the increase in intracellular calcium being the best characterized mechanism [14].

The structure of human IGFBPs is composed of three parts, the N-terminal, a linker sequence, and the C-terminal region. The N-terminal domains of IGFBPs possess several disulfide bonds that contribute to stabilize the structure of the high-affinity IGF-binding sites. The C-terminal has a surface hydrophobic sequence that helps to increase IGF-binding affinity, and in IGFBP3 and IGFBP5 there is an additional basic region implicated in the binding of ALS in serum [15]. The linker domains do not bind IGFs, but they are key regions as they contain several sites for controlled proteolysis, a process that reduces the IGF-binding affinity, resulting in the release of IGFs and an increase in their local concentrations [16]. The bioavailability of IGF1 can also be modulated by N-terminal cleavage of the molecule, producing a tripeptide, glycine-proline-glutamate and a dipeptide, cyclic glycine-proline that can modify the interaction properties of IGF-1 to its binding proteins [17].

Proteolysis of an IGF binding protein was first demonstrated in serum from pregnant humans and rodents [18,19,20], with the placenta reported to be the source of this increased activity [21]. Proteolysis of IGFBP3 was shown to reduce its binding affinity for the IGFs and thus increase its bioavailability [22,23,24]. However, these studies spurred controversy as to whether this proteolytic activity is physiologically significant, with IGFBP proteolysis regulating IGF access to its receptors, or is it merely an artifact. It is now generally accepted that this activity is physiologically significant, and indeed, the demonstration that the loss of specific proteolytic activity causes a clinical phenotype, as discussed below, clearly supports this concept.

**Figure 2:**
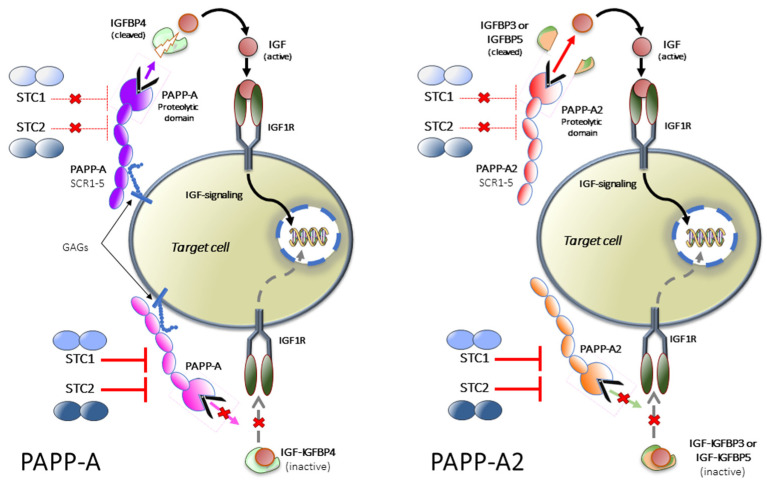
The role of PAPP-A, PAPP-A2, STC1 and STC2 in the IGF system.

The existence of pregnancy-associated plasma proteins (PAPP-A and PAPP-A2) and stanniocalcins (STC1 and STC2) (Figure 2), has been known for more than four decades; however, their regulatory functions in the GH-IGF system were discovered much later. PAPP-A and PAPP-A2 are metalloproteinases that regulate the bioavailability of IGFs. PAPP-A modulates IGF actions through proteolysis of IGFBP2, IGFBP4 and IGFBP5, with IGFBP4 proteolysis and alteration of its binding properties being the main function of this metalloproteinase [25]. In contrast, PAPP-A2 regulates IGF1 bioavailability through cleavage of IGFBP3 and IGFBP5 and *Pappa2* knockout mice present growth retardation, together with high circulating levels of total IGF1 but low free IGF1 levels [26].

In mice, the overexpression of *Pappa* in bone is associated with increased bone mineral density (BMD). On the other hand, the expression of a protease-resistant IGFBP4 inhibited bone growth and reduced BMD, indicating that PAPP-A stimulated bone formation occurs primarily via increasing IGF bioavailability [27]. Likewise, *Pappa* knockout mice are smaller than wild-type littermates, whereas double knockout of *Pappa* and *Igfbp4* lead to an increase in size compared to *Pappa* knockout mice, revealing that cleavage of IGFBP4 by PAPP-A stimulates IGF release to promote growth [28]. Although little is known about the hormonal regulation of PAPP-A, cytokines have been reported to modulate its expression. Indeed, TNF-α and IL-1β promote its expression, whereas interferon-γ inhibits its transcription [29]. The synthesis of this pappalysin seems to occur in a GH-independent manner [30].

The human gene for *PAPP-A* is located on chromosome 9q33.1 and encodes, after post-translational processing, a protein with five domains, three of which appear to be crucial in the actions of this metalloproteinase. The N-terminal domain stabilizes the proteolytic domain that is responsible for cleavage of the IGFBPs and, finally, a region with five copies of complement control protein (CCP), implicated in the binding of PAPP-A to glycosaminoglycans (GAGs) of the cell surface, can increase IGF signaling by releasing IGFs in the proximity of their receptors [31]. In the proteolytic domain of PAPP-A, there are two structural motifs, a zinc-binding consensus sequence that coordinates the catalytic zinc ion in the active site and a methionine residue, important for the structural integrity of the active site. Hydrolysis, initiated in the glutamate residue of the consensus sequence, activates a water molecule bound by zinc, also ligated by three histidines. Proteolysis of IGFBP4 takes place in the link domain, between the Met135 and Lys136 residues, producing two fragments with reduced affinity compared to the intact molecule [32]. This process is IGF-dependent for IGFBP2 and IGFBP4 and IGF-independent for IGFBP5 [33]. The IGFs do not act as cofactors, but they induce a conformational change that adapts the interaction with PAPP-A and allows the cleavage site in IGFBP-4 to be more efficiently accessed [34]. The presence of two calcium-binding Lin12-Notch repeat (LNR) sites in the proteolytic domain, as well as a third in another domain of the molecule, determine PAPP-A specificity and is essential for the proteolysis of IGFBP4, but not for that of IGFBP5. Thus, deletion of the LNR modules provokes the total loss of proteolysis of IGFBP4 but the proteolytic activity toward IGFBP5 is retained [35]. The sequence of *PAPP-A* is different from that of other metzincin members, such as matrix metalloproteinases, a desintegrin and metalloproteinases (ADAMs) and ADAM proteases with thrombospondin motifs [36], and thus constitutes the first member of the pappalysin subfamily. 

Less is known about the second and last member of this subfamily, PAPP-A2, which shares 45% homology of its residues and the three LNR and five motifs responsible for the enzymatic activity with PAPP-A. In addition, the zinc binding site and the methionine residue are conserved in this metalloproteinase. The *PAPP-A2* gene is located on chromosome 1q25.2 and the encoded protein preferentially cleaves IGFBP5 at the Ser143-Lys144 bond, with lower proteolytic activity for IGFBP3. Unlike PAPP-A, PAPP-A2 is present in a soluble form and its cleavage of IGFBPs is IGFs-independent [37]. The specificity of PAPP-A2 for IGFBP3 and IGFBP5 is important since the main pool of circulating IGFs is found in ternary complexes with ALS and IGFBP3 or IGFBP5. This aspect will be discussed later in this review, considering the effects of mutations in *PAPP-A2* on the peripheral IGF axis and its relationship with alterations of human growth.

In the circulation, PAPP-A2 is mostly found in an heterotetrameric structure composed of two PAPP-A subunits and two preforms of eosinophil major basic protein (proMBP). The heterotetramer proMBP is formed via cysteine residues in PAPP-A close to the active site and limits the proteolytic activity of PAPP-A [38]. Other proteases that can cleave IGFBPs, such as kallikreins, cathepsins and matrix metalloproteinases, can enhance IGF1 actions by decreasing the effects of IGFBPs [39]. Whether these proteases compensate from the loss of PAPP-A2 activity in PAPP-A2 deficient patients is unknown. As these patients have low free IGF1 levels and high concentrations of circulating intact fractions of IGFBPs, suggests that if there is compensation of proteolytic activity at the cellular/tissue level it is not reflected in the circulation.

The existence of STCs, inhibitors of pappalysin (Figure 2), has been known for decades and their name is derived from the corpuscle of Stannius, a gland associated with the kidney of fish and involved in calcium regulation [40]. STC1 is a disulfide-bound homodimeric glycoprotein, widely expressed in mammals and in humans. Its gene is located on chromosome 8p21.2. It strongly inhibits PAPP-A cleavage of IGFBP4 and efficiently antagonizes PAPP-A mediated IGF1R activation [41]. In addition, PAPP-A2 can also be inhibited by this STC (Figure 2). The inhibition of pappalysin activity does not require the formation of a covalent complex and is reversible. *Stc1* knockout mice do not show growth defects, while *Stc1* overexpression provokes a severe reduction in postnatal growth [42]. Inhibition of PAPP-A by STC1 prevents the release of IGF1 in the proximity of the growth plate and its interaction with IGF1R, reducing longitudinal bone growth. Indeed, using an organ culture system to assess the effects of STC1 on growth plate chondrogenesis, recombinant human STC1 was shown to block metatarsal growth, proliferation, hypertrophy/differentiation of chondrocyte in the growth plate, and extracellular matrix synthesis [43].

The insulin-like growth factors (IGFs) circulate bound to high-affinity IGF-binding proteins (IGFBPs). IGFBP4, bound to IGF, can be cleaved by the proteinase pregnancy associated plasma protein-A (PAPP-A) to liberate IGF, which can then activate its receptor (IGF1R). Stanniocalcin 2 (STC2) inhibits PAPP-A proteolytic activity through covalently binding to the proteinase, thus inhibiting the release of IGFs. STC1 is also inhibitory but binds PAPP-A reversibly with very high affinity. PAPP-A2 has proteolytic activity toward IGFBP3 and IGFBP5. Both STC1 and STC2 can also inhibit the activity of PAPP-A2, blocking the release of IGFs from IGFBP3 and IGFBP5. Figure modified from Jepsen et al. (2015). GAGs: glycosamino glycans.

The human *STC2* gene is located on chromosome 5q35.1 and encodes a homodimeric glycoprotein. The knowledge of a role of this molecule in longitudinal growth preceded the discovery of its inhibitory actions on the proteolytic activity of PAPP-A and PAPP-A2. Overexpression of STC2 was reported to cause a reduction in size, whereas *Stc2* knockout mice grew more than wild-type littermates [44]. STC2 is a powerful inhibitor of pappalysin activity through the formation of a covalent complex with pappalysins. Inhibition of PAPP-A-mediated proteolysis requires the formation of a disulfide bond with Cys120 of STC2 being implicated in this process [45]. In genome-wide association studies (GWAS), carriers of a rare *STC2* gene missense variant were taller than non-carriers. In vitro functional characterization of this variant showed that its presence leads to decreased binding of STC2 to PAPP-A, resulting in lower inhibition of PAPP-A activity and augmented cleavage of IGFBP4 [46]. One of the most relevant functions of STC2 appears to be the control of PAPP-A activity in tissues by modifying the equilibrium between active and inhibited PAPP-A. Thus, increased STC2-PAPP-A complexes can reduce local liberation of IGFs and subsequently, intracellular signaling [29]. Further understanding concerning how changes in the circulating levels of STC2 impact on free IGF1 is necessary, since transgenic [47] or *Stc2* knockout mice [44] do not present any alterations in circulating IGF1, suggesting that the main function of this stanniocalcin could be to regulate IGF signaling at the cellular level.

## 2. Mutations in the Human *PAPP-A2* Gene

**Figure 3:**
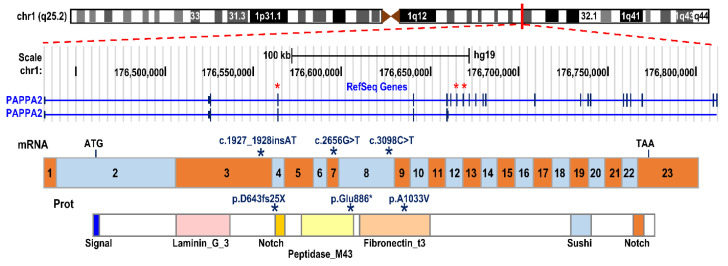
*PAPPA2* gene mutations described to date. Schematic representation of the genomic structure of the *PAPPA2* gene on chromosome band 1q24, the encoded mRNA and the protein with its relevant functional domains. The stars indicate the location in the exons, mRNA and protein where the subjects’ mutations are found.

To date, three pathogenic variants, in three unrelated families from different geographic origins, have been described. The first variant was described in a Spanish family harboring a frameshift mutation (c.1927_1928insAT; p.D643fs25*) in exon 3 of the *PAPP-A2* gene, with two homozygous siblings being affected [48]. The second variant was found in a consanguineous Palestinian family, with three siblings carrying a missense variant (c.3098C > T; p.Ala1033Val) in exon 8 [48], and the most recently described variant was found in a family from Saudi Arabia, with two affected brothers expressing a nonsense variant (c.2656G > T; p.Glu886*) in exon 7 of the *PAPP-A2* gene [49] (Figure 3). 

In total, seven homozygous patients with very similar clinical, auxological and biochemical hallmarks have been reported. Serum concentrations of PAPP-A2 concentrations in these patients were very low or undetectable. In the first and third families, homozygous for the D463fs25* and p.Glu886* variants, respectively, circulating PAPP-A2 levels were undetectable. In contrast, in the second family PAPP-A2 concentrations were detectable but in the low end of the normal range. The p.Glu886* variant codes for a truncated protein were classified as likely pathogenic according to the criteria of the American College of Medical Genetics. In the first two cases, in vitro functional studies were performed, demonstrating that neither of the variants could cleave the IGFBPs [48].

### 2.1. Clinical Phenotype

This syndrome, where there is a lack of PAPPA2 activity, is characterized by: (i) progressive postnatal growth retardation; (ii) reduced or absent circulating levels of PAPPA2; (iii) considerably elevated serum concentrations of total IGF1, IGF2, IGFBP3, IGFBP5, and ALS, together with very low free IGF1 levels and IGF bioactivity; (iv) thin long bones; and (v) decreased bone mineral density.

The absence of PAPP-A2 proteolytic activity in these patients results in an increase in circulating ternary complexes, together with very low free and bioactive IGF1 levels. It is possible that the reduction in IGF1 bioactivity would diminish its negative feedback effect on somatotrophs in the pituitary, as well as at the hypothalamic level, resulting in increased GH production and secretion, as observed in these patients. This increase in GH secretion would, in turn, stimulate the production of more IGF1, IGFBP3, IGFBP5 and ALS in the liver, resulting in the formation of more ternary complexes. Indeed, total IGF1, IGFBP3 and ALS concentrations are markedly increased in all patients. However, serum IGF2 and IGFBP5 levels were increased in the first two families but not in the Saudi subjects. These differences are of great interest as they could indicate differential-selective proteolytic activity based on the genetic mutation, although this supposition requires further investigation and the identification of more affected individuals. As these patients lack PAPP-A2 proteolytic activity, the ternary complexes accumulate in the circulation. Indeed, when analyzed by neutral size-exclusion chromatography, the level of these complexes was shown to be significantly higher in the patients of the Spanish family, when compared to their unaffected siblings, as well as to prepubertal control levels [48]. One might speculate that the apparently normal growth velocity and height in these patients during their early prepubertal years is because the accumulation of these ternary complexes is gradual, and they have not yet reached sufficient levels to impede the function of this axis. It is also possible that during these early years the control of this axis, in particular the mechanisms by which IGFs are liberated, differ from those in the later stages of prepubertal and pubertal growth. This hypothesis deserves future investigation.

### 2.2. Auxology in PAPP-A2 Deficient Patients

A key clinical feature of PAPP-A2 deficiency is the progressive appearance of harmonic postnatal growth retardation. Indeed, patients with inactivating mutations in the *PAPP-A2* gene present a growth pattern that differs from the rest of the known causes of short stature associated with alterations in the GH axis, although the number of patients and the information available regarding their auxologic pattern is limited. The short stature of these patients is of postnatal origin with the effect on prenatal growth not sufficiently clear to date. Indeed, two of the patients (siblings) had a weight and length at birth above the mean for their gestational age, while 4 patients had criteria for Small for Gestational Age (SGA) (weight and/or length at birth ≤−2 SDS for their gestational age). This finding is not surprising if we consider that IGFs, independently of GH, play a decisive role in fetal growth.

More surprising is the postnatal growth pattern of these patients, which is characterized by a progressive decrease in growth velocity, resulting in a progressive deterioration of their height that ultimately results in a short adult height. The time of onset (usually between 2 and 6 years of age) and the severity of the deterioration in height vary from one patient to another, ranging from mild forms, which may initially be confused with normal variants of short stature, to severe short stature. However, even when their height and growth velocity are apparently normal, their growth lane on the growth curve is below that which corresponds to their family context and at the time of diagnosis all patients were quite discordant with their mid-parental height. This emphasizes the fact that height should always be evaluated within the family context and that further investigation of these patients is needed if their height is quite distant from their target height [50,51]. Moreover, it is of utmost importance to monitor deviations over time in growth velocity as this lack of PAPP-A2 activity results in a progressive decrease in growth velocity, most likely due to the gradual accumulation of ternary complexes and decreases in IGF1 bioavailability.

The rate of bone maturation does not appear to be significantly altered and puberty usually begins at a normal age, with a greatly diminished or even absent pubertal growth spurt that results in an even further deterioration of adult height. However, the auxological data available regarding pubertal growth in these patients and final height are very limited due to the reduced number of patients identified to date, as well as the lack of precise data in some of these patients. The variability in clinical expression could be due in part to the type of mutation and the degree to which the proteolytic action is altered, but also to other genetic and environmental factors that modify the disease manifestation. 

In patients with PAPPA2 deficiency the appearance of clinical signs resembles that observed in other inborn errors of metabolism, where during early life there is an apparently symptom-free interval that later deteriorates unexpectedly. The precise underlying pathophysiological mechanisms responsible for the peculiar growth pattern observed in these patients that results in the progressive evolution of short stature are unknown. However, as stated above, the gradual accumulation of trimolecular complexes concomitantly with a slow continuous decrease in the amount of free IGFs available could explain this phenomenon. Nonetheless, there is very little known regarding the changes in the concentrations of these complexes during development. It is important to note that the accumulation of trimolecular complexes does not appear to have toxic effects, but the more long-term effects of this situation in these patients remain to be determined.

Following the Kalberg three-component curve for growth [52], the first component of the curve (infantile) that encompasses the fetal period and the first 2–3 years of life, seems to be the least affected in these patients, possibly because the infantile growth initially depends mainly on nutrition during the first year of life; thereafter, growth depends on both the GH/IGF axis and nutrition. The second component of the curve, or the prepubertal period, which under normal conditions is dependent on genetic components and regulated mainly by the GH-IGF axis, is when the growth of these children appear to experience a progressive deceleration. Puberty, the third component of the curve, does not appear to be significantly delayed, but the growth spurt normally associated with this developmental stage is decreased because of its dependence on the synergistic actions of the GH axis and sex steroids on the growth plate. Pubertal onset does not appear to be affected in this entity, as all affected patients displayed spontaneous pubertal development. Nonetheless, more subjects are necessary to draw clear conclusions regarding puberty, or other possibly reproductive affections.

### 2.3. Bone and Teeth Abnormalities

No signs of skeletal dysplasia have been observed in any of the patients, except for the presence of thin long bones, as well as small chins and moderate microcephaly. In the subjects that have been analyzed by DXA, mild osteoporosis or osteopenia have been observed, suggesting that bone formation/turnover is affected. Studies of body composition have shown a reduction in lean mass with a high percentage of body fat [53]. 

**Figure 4:**
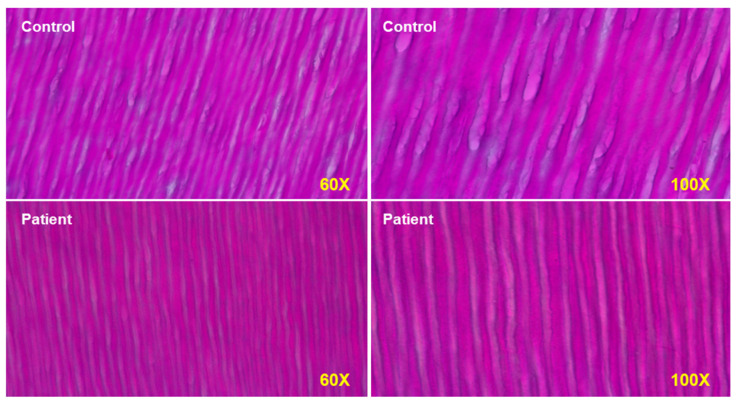
*PAPP-A2* null patient has narrower dentin tubules at seen at magnifications of 60× and 100×. Both odontoblasts and amyloblasts have impaired function in the patient.

The effects of PAPP-A2 deficiency on bone cannot be directly addressed in the affected human subjects for obvious reasons. Mouse models of *Pappa2* mutations or deletion revealed growth retardation (decreased linear growth) but reported different findings regarding sex or age-dependent effects on BMD. In human subjects, however, it is possible to take advantage of another mineralized tissue, the tooth, that can indirectly reflect some processes occurring in bone. The micro-CT analysis of a tooth from one of the patients showed significantly decreased enamel and dentin density [48]. A molar obtained from the Spanish female subject with PAPP-A2 deficiency also revealed abnormal enamel prisms with significantly reduced cross striata (Figure 4), reflecting a 24 h circadian rhythm of enamel deposition during development. These findings possibly indicate an impaired mineralization process, and although indirect, evidence from teeth of affected individuals could help to understand how PAPP-A2 regulates tissue mineralization, as these hallmarks are probably a consequence of the lack of free IGF1, as this growth factor is also important in tooth development.

### 2.4. Glucose Metabolism

Disturbance in carbohydrate metabolism have also been observed in patients with PAPP-A2 deficiency, including mild basal hyperinsulinemia, insulin resistance and glucose intolerance. All patients with homozygous mutations in *PAPP-A2*, showed basal hyperinsulinemia of unknown etiology, but this may be related to the increase in GH. They also had normal fasting glucose and decreased IGFBP1 and IGFBP2, with reduced IGFBP1 levels, having been associated with hyperinsulinemic/insulin-resistant states [54]. Moreover, both IGF1 and IGF2 have been implicated in glucose metabolism, and alterations in their levels and functions could also be involved in the changes in insulin levels and glucose and insulin tolerance, although studies to confirm this must be performed. Notably, glucose abnormalities and hyperinsulinemia have been reported in other alterations of the GH-IGF axis such as IGFALS deficiency [55], and these are most likely due to elevations in GH secretion and the normal physiological balance of the GH/IGF axis being disrupted.

### 2.5. Treatment

Treatment of PAPP-A2 deficient patients with recombinant human IGF1 (rhIGF1) improves growth [56,57], but not GH treatment as it was shown to be ineffective in one patient [49]. This is probably because GH would simultaneously increase the three components of the trimolecular complex, IGFs, IGFBPs and ALS, which would not only result in an increase in the free fraction of IGFs but would contribute to further increase the levels of sequestered IGFs.

In the Spanish siblings, treatment with rhIGF1, subcutaneously twice daily, was initiated when they were still prepubertal. An increase in growth velocity was evident in both the short- and the long-term, reaching their midparental target height, with an increase in BMD and modifications in body composition [56,57]. Laboratory analyses revealed a decrease in spontaneous GH secretion and an increase in free IGF1 values following rhIGF1 administration. No side effects have been observed to date, including no episodes of hypoglycemia while under continuous glucose monitoring.

The youngest patient from the Saudi Arabian family was also under treatment with rhIGF1 [49]; however, a modest growth response was reported, most likely as a result of the limited duration of the treatment so far. In consequence, as recombinant PAPP-A2 is currently unavailable for treatment, rhIGF1 should be considered a safe and effective therapy in patients with PAPP-A2 deficiency to increase their growth and BMD, especially during childhood and puberty.

## 3. PAPP-A2 Mouse Models: What Are We Learning?

While a few controversies exist, many of the findings obtained from studies using knock-out (KO), knock-in (KI) and knock-down (KD) mouse models of *Pappa2* recapitulate the main features described in patients with *PAPP-A2* mutations [58]. In this section, we summarize findings from mouse models of *Pappa2* with a special focus on auxological, hormonal, metabolic and BMD alterations during postnatal growth and adulthood (Table 1). Finally, we propose studies of experimental treatments using recombinant murine Igf1 (rmIgf1) and recombinant murine Pappa2 (rmPappa2), in mouse models with Pappa2 deficiency, which could help to provide information regarding their ability to palliate growth retardation, as well as the effects on other physiological processes such as pubertal development, metabolism, and bone formation and quality, and possible long-term complications. 

### 3.1. Expression Pattern of Pappa2 in Mice

In mammals [59,60,61], Pappa2 is highly expressed in the feto-maternal interface of the placenta at all gestational stages, in addition to being expressed in specific locations in other tissues throughout the body of the embryo and adult mouse [26,62,63]. Christians et al. [62] reported high expression of Pappa2 in the stomach and skin of adult mice, as well as in murine embryos, and lower expression in the kidney, brain, heart, lung, testis, pancreas and prostate gland. Later, Conover et al. [26] indicated that, in addition to the elevated expression of Pappa2 in mouse placenta and embryo, it is also highly expressed in the prostate, colon, lung, ovaries, tibia, brain, spinal cord, testes and kidneys, while being undetectable in the spleen, soleus, adipose tissue, thymus, uterus, liver, skin and heart [26]. The importance of Pappa2 in bone and joint development is emphasized by the demonstration of its expression in the epiphyseal cartilage of the acetabulum and femoral head of the hip joints of rats at different perinatal stages [64], as well as in the epiphysis and metaphysis, including osteoblasts, of the femur and tibia of 19-day-old mice [65]. Analysis of Pappa2 expression has also been performed in zebrafish embryos, larvae and adults, with adult tissues such as bone, muscle, eye, brain, gill, kidney, gastrointestinal tract, ovary and testis shown to have higher expression levels than other tissues [66]. Together, these findings confirm the physiological importance of PAPP-A2 in the promotion of placental, fetal and postnatal growth, and support the hypothesis that PAPP-A2 may act as a local tissue-specific modulator of IGF1 bioavailability by cleavage of ternary complexes containing IGFBP3 or IGFBP5 and IGFALS.

### 3.2. Available Models and Their Differences in Growth and Body Weight

Two knock-out and one knock-in mouse models with targeted disruption of *Pappa2* have been studied to date [26,67,68]. The most striking phenotype of these genetically modified mice is postnatal growth retardation with low levels of free Igf1 and high levels of total Igf1 [58], similar to that seen in PAPP-A2 deficient patients. The first homozygous mice with a constitutive *Pappa2* deletion were developed and described by Conover and colleagues [26]. In this study, 16-week-old *Pappa2* KO males weighed 10% less and were modestly smaller on average, but this was not significantly different from male wild-type (WT) littermates. In contrast, *Pappa2* KO females had significantly lower body weight (30%) and length (10%), which was associated with disproportionally larger organs, expressed as wet weight relative to total body weight, including heart, spleen, liver, kidneys and brain. Two years later, the second constitutive *Pappa2* KO mouse model was reported [68] resulting in further insights into postnatal growth retardation. These authors did not detect any effect of constitutive *Pappa2* deletion on placental or embryonic mass or on the dry weights of stomach, spleen, pancreas, kidneys, liver, lungs or heart of *Pappa2* KO mice at 10 weeks of age. However, they described reductions in both body weight and tail length at 3, 6 and 10 weeks of age [68]. Subsequently, these authors developed genetically modified mice by disrupting *Pappa2* in SP7-expressing osteoblasts (conditional *Pappa2* deletion) and reported a sex-independent decrease in body weight and tail lengths at all ages [65]. These results indicated that the effect of whole body *Pappa2* deletion was greater than the effect of osteoblast-specific *Pappa2* deletion. In contrast, when specific *Pappa2* deletion was triggered at 20 weeks of age by tamoxifen-induced *Cre*-mediated recombination (resulting in whole body *Pappa2* deletion), no difference between genotypes in body weight was detected [69]. Recently, using the second mouse model for constitutive *Pappa2* deletion, our research group also reported a sex-independent reduction in body length of *Pappa2* KO mice at 8 months of age [70]. A constitutive knock-in mouse model employing the human p.Ala1033Val mutation in *PAPP-A2* was generated using the CRISPR/Cas9 system [67]. These human *PAPP-A2* KI mice, expressing PAPP-A2 proteins that lack protease activity for IGFBP3, confirmed the growth retardation associated with the p.Ala1033Val missense mutation previously described in humans [48]. These mice showed reductions in body length and weight in both males and females (89.6% and 77.7%, respectively, of mean body weight with respect to WT littermates) when they were measured from 4 to 16 weeks of age. These authors also report a proportionally higher fat mass and lower lean mass in *PAPP-A2* KI mice of both sexes. However, liver weight relative to total body weight was only elevated in female human-*PAPP-A2* KI mice at 16 weeks of age.

### 3.3. Differences in Hormonal Alterations in Available Models of Pappa2 Deficiency

**Figure 5:**
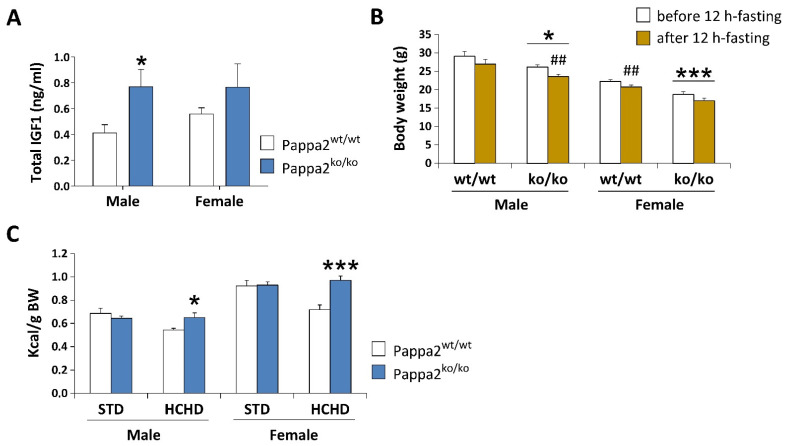
Analysis of plasma concentrations of total IGF1 (**A**), body weight (BW) before and after 12 h-fasting (**B**), and caloric intake relative to BW after 3 week-feeding of a standard (STD: 2.9 kcal/g) or a high carbohydrate (HCHD: 3.85 kcal/g) diet (**C**) in constitutive Pappa2 KO mice of both sexes at 8 months old. Data are represented as mean ± S.E.M. (*n* = 7–8/group). Tukey-corrected tests: *^/^*** *p* < 0.05/0.001 versus respective Pappa2^wt/wt^ mice; ^##^ *p* < 0.01 versus mice before 12 h-fasting.

In the model presented by Conover and colleagues [26], no changes in Pappa2 mRNA levels or Igfbp5 proteolysis in fibroblasts of *Pappa2* KO embryos were found. However, at 4 months of age, these *Pappa2* KO mice had a dramatic reduction (95%) in free Igf1 concentrations and elevated concentrations (50%) of total Igf1, supporting functional *PAPP-A2* defects in the release of IGF1 from its ternary complex, as observed in affected patients [48]. Christians and colleagues [69] also reported an increase (60%) in circulating levels of total Igf1 at 1.5 months of age in their *Pappa2*-KO model. Recently, these authors described a reduction in the circulating levels of a putative free bioactive Igf1 in 2-month-old *Pappa2* KO mice [71]. Circulating free Igf1 is also decreased and total Igf1 increased in the *Pappa2* KI mice at 4 months of age [67]. Using the second *Pappa2* KO mouse model developed by Christians et al. [68], we have observed higher circulating levels of total Igf1, specifically in males, but not in *Pappa2* KO females at 8 months of age (Figure 5A). At 1.5 months of age constitutive *Pappa2* KO mice also had 2-fold higher levels of Igfbp5 and 15-fold lower levels of Igfbp3 [69]. Increased circulating levels of Igfbp5, recently confirmed in 2-month-old *Pappa2* KO mice, were not influenced by gestation and recovery after lactation [71], with these variations also continuing to be observed in the plasma of older mice with a mean age of 7 months [69]. However, at 7 months of age, no effect of *Pappa2* deletion was observed on mRNA levels of *Igfbp3* or *Igfbp5* in the liver or kidney, but Igfbp5 protein levels were increased in ovaries of female *Pappa2* KO mice [72]. In contrast, osteoblast-specific deletion of *Pappa2* did not alter circulating levels of Igfbp5 in mice at 18–19 days of age [65], suggesting a local Pappa2-Igfbp5 mechanism for Igf1 function in bone physiology not yet addressed. When whole body *Pappa2* deletion was induced at 5 months of age, no difference in circulating levels of Igfbp5 was found between genotypes, but lower levels of circulating Igfbp3 were detected in a body size and sex-independent manner [69]. In contrast, increased circulating levels of Igfbp3 and Igfals were found in *Pappa2* KI mice at 4 months of age [67]. We found a genotype effect on *Igfbp3* mRNA levels in the bone of 8-month-old *Pappa2* KO mice with female *Pappa2* KO mice having higher *Igfbp3* mRNA levels in the tibia than female WT littermates [70]. 

Analysis of a morpholino-mediated *Pappa2* KD zebrafish embryo model (50% and 43% overall sequence identity to human *PAPP-A2* and *PAPP-A*, respectively, and conserved proteolytic activity in human IGFBP3 and IGFBP5) suggested that Pappa2 actions in normal growth and development may also be exerted through non-proteolytic modulation of Notch signaling [66]. While IGFBP-independent mechanisms of PAPP-A2 cannot be excluded, overall results to date indicate that the modulation of IGF1 bioavailability through its release from ternary and binary complexes should be further investigated in a tissue, sex and age-specific manner.

### 3.4. Metabolic Disturbance in Available Models of Pappa2 Deficiency

Despite the smaller size and lower weight of *Pappa2* KO mice at 3, 6, 10 and 14 weeks of age in the model described by Christians and colleagues [69], disruption of circulating Igf1 levels did not affect glucose tolerance, body weight gain or adiposity at 6 weeks of age when constitutive *Pappa2* KO mice were fed a high-fat diet (45% fat) from 17 to 25 weeks of age. Likewise, using the mouse model developed by Christians et al. [68], we found no modifications in glucose tolerance in constitutive *Pappa2* KO mice at 2 months of age, but we found that these *Pappa2* KO mice lost more body weight than their WT littermates when fasted for 12 h (Figure 5B). In contrast, *Pappa2* KI mice are reported to be glucose intolerant and insulin resistant [67]. These discrepancies in glucose handling may be due to differences in the dietary supply of glucose, amino acids and lipids to the liver, in addition to metabolic compensation, such as an increase in insulin concentrations and/or decrease in the insulin-induced autophosphorylation of its receptor and insulin receptor substrate, observed in constitutive *Pappa2* KO mice that may not be produced in human *Pappa2* KI mice due to the presence of a Pappa2 protein, at least in some patients, despite its lack of protease activity. In our preliminary studies, we did not observe differences in body weight gain compared to WT when *Pappa2* KO mice were fed a high-carbohydrate diet (HCHD, 70% sucrose and fructose) for 3 weeks, but they increased their caloric intake (relative to body weight), suggesting higher energy expenditure associated with lower Igf1 availability when exposed to a high carbohydrate diet (Figure 5C). Because diabetes and obesity induced by hypercaloric diets have been associated with impairments in bone mineral density [73,74,75], future studies should evaluate whether deficiency in Igf1 bioactivity impacts energy homeostasis, regulating bone mass in a sex and diet-specific manner [76].

### 3.5. Variations in Allometric and Bone Mineral Alterations in Mouse Models of Pappa2 Deficiency

Allometric measurements in the whole body *Pappa2* KO mouse developed by Conover and colleagues [26] indicated that femur length of adult mutant mice, expressed as a percentage of total body length, was not significantly different compared to WT mice. These authors also found no evidence of developmental delay in embryo length or bone mineralization. In contrast, Christians and colleagues [68] reported that their whole body *Pappa2* KO mouse model had defects in the size of the mandible, as well as reduced lengths of the skull, humerus, femur, tibia, pelvic girdle and tail bone at 10 weeks of age. These authors also reported genotype effects on femur length of *Pappa2* KO mice at 19 and 30 weeks of age [77]. Osteoblast-specific *Pappa2* deletion also affects bone dimensions, including mandible, skull, humerus, radius, femur, tibia and pelvic girdle [65]. Moreover, when the effects of whole body and osteoblast-specific *Pappa2* deletion were compared, only mandible dimensions and femur length showed significant differences, emphasizing the importance of PAPP-A2 at the tissue level. Studies of the *Pappa2* KD zebrafish model suggested that Pappa2 function is required for normal development of Meckel’s and cranial cartilages [66]. 

We found sex-specific reductions in proportional lengths and weights of the femur and tibia (relative to bone weight and body weight) of *Pappa2* KO mice at 8 months of age [70]. The role of the GH/IGF1 axis in sex differences in bone growth is still under question and some data are difficult to interpret [78]. In males, the androgen-induced increase in GH is correlated with an increase in IGF1. In females, the estrogen-induced increase in GH is sometimes accompanied by a decrease in IGF1 [79]. This leads to the hypothesis that in early puberty sex steroids are stimulatory to periosteal bone growth through the GH/IGF1 axis, while in late puberty high levels of estrogen in females are inhibitory to bone formation [80]. In this regard, the main mechanisms by which aromatase activity and sex steroids influence bone growth [81], including longitudinal bone growth, attainment of peak bone mass, the pubertal growth spurt, epiphyseal closure, and normal bone remodeling, should be evaluated in *Pappa2* KO mice during puberty.

Christians and colleagues [68,77] also reported non-size dependent differences in the shape of the pelvic girdle (ischium shape, pubis length and ilium width) and mandible (smaller distance between the tips of the angular and coronoid processes) of whole *Pappa2* KO mice, suggesting that PAPP-A2 deficiency-related disproportionality of pelvic gridle shape may contribute to hip dysplasia in humans and result in difficulties in giving birth [82,83].

When quantitative (micro) computed tomography (QCT) was applied to analyze adult bone phenotype, Conover and colleagues [26] described proportional decreases in area and bone mineral content in cortical and trabecular femur of 16-week-old Pappa2 females only. Christians and colleagues [77] also described sex and age-specific changes in bone structure and mass that differ between trabecular and cortical bone. These authors confirmed reductions in trabecular parameters in whole *Pappa2* KO mice at 10 and 19 weeks of age but, unlike the previous study [26], increases in cortical mineral density were specifically detected in the femur of female *Pappa2* KO mice at 19 and 30 weeks of age, as well as increases in the cortical area fraction of the femur in *Pappa2* KO mice at all ages [77]. In a recent study [84] a genotype effect on trabecular thickness and cortical area fraction recovery after lactation was reported in female *Pappa2* KO mice at 2 and 5 months of age. Abnormalities in bone morphology were also described in the femur of *Pappa2* KI mice at 16 weeks of age [67], with several cortical and trabecular femur traits including total cross-sectional area, bone area, linear bone length and trabecular BMD being decreased in both males and females while, unlike previous studies, cortical bone thickness and mineral density, among other traits, were not affected. These authors suggested that the disproportionality of these bone alterations may be associated with inhibition of periosteal bone growth and a lower robustness in the female bone. Overall, these studies with controversial results in the effects of Pappa2 deficiency on bone structure and mineral density require further investigation to address bone composition, formation, and remodeling. In this regard, we recently described sex-specific changes at 8 months of age in femur composition of the same *Pappa2* KO mouse model. By using X-ray powder diffraction (XRD) and attenuated total reflectance-Fourier transform infrared spectroscopy (ATR-FTIR), male *Pappa2* KO femur was found to have alterations in mineral crystallinity and relative content of bone compounds containing phosphates and carbonates, while female *Pappa2* KO femur had a reduction in collagen maturity only [70]. This study suggested that alteration of femur mineral composition associated with Pappa2 deficiency may denote bone fragility and immaturity.

### 3.6. Effects of Pappa2 Deficiency on Mechanisms Regulating Bone Development and Remodeling

Recent studies have evaluated the contribution of Pappa2 to molecular mechanisms regulating bone formation and resorption [70,77]. Reductions in the serum levels of N-terminal propeptide of type I procollagen (PINP), a marker of osteoblast activity and bone formation, and tartrate-resistant acid phosphatase form 5b (TRACP 5b), a marker of osteoclasts implicated in bone resorption, were detected in constitutive *Pappa2* KO females at 6 and 19 weeks of age [77]. Recently, Rubio and colleagues [70] confirmed that bone turnover was locally disturbed in older *Pappa2* KO. However, unlike in the previous study [77], sex-specific increases in the gene expression of osteocalcin, a marker of bone formation, and osteopontin, a marker of bone resorption, in the tibia of *Pappa2* KO females at 8 months of age were observed [70]. These modifications that would indicate higher bone turnover in female mice are consistent with the increased rate of bone loss and osteoporosis risk in women [85]. However, the controversial results regarding the effects of Pappa2 deficiency on circulating and local mechanisms controlling bone remodeling should be further addressed. Indeed, a recent study reported that the hip of new-born rats affected by developmental dysplasia had lower gene and protein expressions of *Pappa2* and *Igf1*, and increased expression of *Igfbp5* [64]. When *Pappa2* expression was knocked-down in the area of the hip of new-born female mice by a direct injection of Cas9/PAPP-A2 sgRNA lentiviruses, the reduced expression of *Pappa2* and *Igf1* downregulation or *Igfbp5* upregulation was suggested to interfere in hip joint development, including reduced fibroblast collagen synthesis and cartilage chondrocyte proliferation [64]. This finding, together with previous data related to disproportionate pelvic growth, suggests that Pappa2 can regulate maternal pelvic size and could be implicated in the developmental dysplasia of the hip and early osteoarthritis [64,68,86].

### 3.7. Experimental Treatments in Mouse Models of Pappa2 Deficiency

Treatment with rhIGF1 was employed to increase growth velocity and height and to palliate bone loss in prepubertal patients with PAPP-A2 deficiency [49,53,56,87]. Moreover, growth velocity continued to increase and bone mineral density was normalized after 6 years of rhIGF1 treatment [57]. Studies have also reported beneficial effects of rhIgf1 on bone formation in aged mice [88] and ovariectomized rats [89]. However, few studies have evaluated the efficacy of Igf1 treatment in mouse models of Pappa2 deficiency that recapitulate the main features described in patients with *PAPP-A2* mutations. We recently provided some insight into the short-term effects of a single administration of rmIgf1 (0.3 mg/kg) on bone composition and remodeling in adult *Pappa2* KO mice [70]. Specifically, rmIgf1 treatment changed mineral compositional properties and increased collagen maturity in the bone of *Pappa2* KO females. These rmIgf1 effects on female bone composition were accompanied by increased mRNA levels of *Igfbp3* and *Igfbp5*, suggesting a feedback response to Igf1 bioavailability and signaling, as previously described by using in vitro assays [24] and in human trials with a long-term treatment of rhIGF1 [57]. This treatment also produced increases in the mRNA levels of *Col1a1* and *osteopontin*, suggesting changes in bone matrix resorption and formation [70]. Overall, these results most likely indicate that the concurrence of higher Col1a1 expression and collagen maturity may underlie bone strength in adult *Pappa2* KO females.

## 4. Future Directions

### 4.1. Future Directions in Animal Models

Further research using the available models of Pappa2 deficiency is clearly required to assess and compare the efficacy of rmIgf1 treatment with different therapeutic strategies, including the classic rmGH and especially the promising rmPappa2, on several unknowns related to growth velocity and bone size from postnatal to pubertal stages, as well as on bone quality from adult to elderly stages, in a sex-dependent manner. The impairments in glucose metabolism and bone mineral density described in patients with PAPP-A2 deficiency suggest that changes in signaling pathways of energy metabolism affected by insulin sensitivity and regulating bone development and remodeling in response to IGF1 treatment should be jointly evaluated as a key approach to better understand the physiological and metabolic derangements related to short stature. We should highlight that no studies to date have reported insights into modifications in the central mechanisms involved in regulation of the GH-IGF axis in the context of PAPP-A2 deficiency. Future studies using rmIgf1 treatment in mouse models of Pappa2 deficiency should also address whether the recovery of Igf1 signaling modulates hypothalamic control of energy balance and bone physiology. Moreover, alterations in GH and IGF1 signaling are well-known to result in a decline in physical and mental function (neurogenesis, microcephaly, mental impairment, psychomotor and behavioral deficits, and hearing loss) in addition to >90% lethality [90,91]. However, the literature to date provides no data in Pappa2 mouse models to support any alteration in brain development, cognition or any other brain function and future studies should address this issue.

### 4.2. Future Directions in Humans

Defects in numerous members of the GH-IGF system and associated mechanisms have been shown to result in short stature, with defects in the metalloprotease PAPP-A2 being one of the latest causes of this condition to be described. Three families with modifications in this protein have been shown to date, but more families are expected to be identified after the description of a specific phenotype has been reported. The development of short stature due to PAPP-A2 deficiency involves the reduction of free IGF, and thus a reduction in IGF activity. The sequestering of the IGFs in ternary and binary complexes clearly underlies these effects. However, there is still much left to be determined regarding the tissue specific effects of this deficiency, as well as the long-term effects. Indeed, longitudinal growth and bone shape and composition are affected, but the IGFs have myriad effects including involvement in metabolism, cancer, and neurodegenerative diseases. How the lack of this specific proteolytic activity affects these processes in the long-term remains to be established [92]. 

It is probable that patients with PAPP-A2 deficiency are underdiagnosed as some patients may present “normal height” in their first evaluation or the physician does not correctly interpret the high IGF1 levels or the decline in growth velocity that these patients present during childhood. It is necessary to determine serum PAPP-A2 levels in children with short stature and elevated serum IGF1 levels. If circulating PAPP-A2 levels are very low or undetectable, the *PAPP-A2* gene should be sequenced. In our personal experience, although limited, treatment with rhIGF1 improves longitudinal growth in patients with PAPP-A2 deficiency and this should be started as early as possible. Until recombinant PAPP-A2 is available for clinical trials, we cannot speculate on its potential growth-promoting effects.

## Figures and Tables

**Table 1:** Summary of the main effects of Pappa2 deficiency on auxological, hormonal, metabolic and bone mineral properties.

	Constitutive *Pappa2* KO	Constitutive *Pappa2* KO	Constitutive Induction of *Pappa2* KO	Conditional *Pappa2* KO in Osteoblasts	Constitutive Human *PAPP-A2* KI
**Main references**	*Conover* et al. *2011*	*Christians* et al. *2013*, *2015a*, *2019*; *Rogowska* et al. *2021*; *Rubio* et al. *2021*	*Christians* et al. *2015a*	*Amiri and Christians, 2015*	*Fujimoto* et al. *2019*
**Auxological parameters**					
**Body weight (BW)**	Ns ^1^ in males Reduction in females	Reduction	ns	Reduction	Reduction
**Body length**	Ns in males Reduction in females	Reduction	--	Reduction in tail	Reduction
**Organ size**	Ns in males Increase in females	ns	--	--	Increase in liver
**Body composition**	--	--	--	--	Higher fat mass. Lower lean mass
**Hormonal parameters**					
**Free Igf1**	Decrease	Decrease	--	--	Decrease
**Total Igf1**	Increase	Increase	--	--	Increase
**Igfbp5**	--	Increase in plasma Ns in liver and kidney Increase in ovaries Ns in tibia	ns	ns	--
**Igfbp3**	--	Decrease in serum Ns in liver and kidney Increase in tibia	Decrease	--	Increase
**Igfals**	--	Ns in tibia	--	--	Increase
**Energy metabolism**					
**Glucose tolerance**	--	ns	--	--	Intolerant
**Insulin sensitivity**	--	ns	--	--	Resistant
**Adiposity**		ns	--	--	--
**BW loss**	--	Increase in fasting	--	--	--
**Caloric intake**	--	Increase in HCHD ^2^	--	--	--
**Allometric parameters**					
**Femur length**	ns	Reduction	--	Reduction	Reduction
**Femur weight**	--	Reduction	--	--	--
**Other bone dimensions**	--	Defects	--	Defects	--
**Bone shape**	--	Defects (pelvic girdle and mandible)	--	Defects (pelvic girdle and mandible)	--
**Bone mineral properties**					
**Bone mineral content**	Decreases in trabecular and cortical femur	Decrease in trabecular femur Increase in cortical femur	-- --	-- --	Decrease in trabecular femur Ns in cortical femur
**Bone mineral composition**	--	Alterations in male femur	--	--	--
**Collagen maturity**		Decrease in female femur	--	--	--
**Bone remodeling**					
**Bone formation and resorption**	--	Decreases in female serum Increases in female tibia	--	--	--

^1^ Ns, not significant; ^2^ HCHD, high carbohydrate diet.
